# Physicochemical Properties and Tissue Structure of High Kernel Elongation Rice (*Oryza sativa* L.) Varieties as Affected by Heat Treatment

**DOI:** 10.3390/foods12112207

**Published:** 2023-05-31

**Authors:** Anna Arina Bt Ab. Halim, Mohd Y. Rafii, Mohamad B. Osman, Samuel C. Chukwu, Yusuff Oladosu

**Affiliations:** 1 Laboratory of Climate Smart Food Crop Production, Institute of Tropical Agriculture and Food Security, Universiti Putra Malaysia (UPM), Serdang 43400, Selangor, Malaysia; adrinafashlina90@gmail.com (A.A.B.A.H.); chukwu.samuel@ebsu.edu.ng (S.C.C.); oladosuy@upm.edu.my (Y.O.); 2 Department of Crop Science, Faculty of Agriculture, Universiti Putra Malaysia (UPM), Serdang 43400, Selangor, Malaysia; 3 Malaysia Agriculture Research and Development Institute (MARDI), Serdang 43400, Selangor, Malaysia; mbopar2004@yahoo.com; 4 Department of Crop Production and Landscape Management, Faculty of Agriculture and Natural Resources Management, Ebonyi State University, Abakaliki PMB 053, Nigeria

**Keywords:** ageing, apparent amylose, absolute amylose, amylopectin, scanning electron microscope (SEM), gel-permeation chromatography (GPC), branch chain length distribution

## Abstract

Heat treatment could affect the structure and properties of rice varieties. The present study was conducted in order to determine the effects of heat treatment on the physicochemical properties and tissue structure of Mahsuri Mutan, Basmati 370 and MR219 rice varieties. The three rice varieties were subjected to heat treatment (ageing) at 90 °C, using an oven, for 3 h. After the heat treatment, the samples were cooled at room temperature (25 °C) for 1 h. Physicochemical properties, such as alkali digestion value, water uptake ratio, solids in cooking water, high kernel elongation ratio and amylose contents, were determined. The procedure used in determining both apparent and absolute amylose involved measuring the iodine affinity of defatted whole starch. Ahigh-performance anion-exchange chromatograph was used to analyse branch chain length distribution of amylopectin quantitatively. The starch structure of the rice samples was observed under a scanning electron microscope. Data collected on physicochemical traits, heat treatment and control (ageing and non-ageing) were subjected to an analysis of variance using SAS software version 9.4. In this study, Mahsuri Mutan and Basmati 370 showed superior high kernel elongation as compared to their respective rice progenies. This study also found that heat treatment directly affected the increasingly high kernel elongation for both populations. The phenotypic correlation co-efficient indicated that there was a high positive correlation between high kernel elongation and water uptake ratio, implying that selection for water uptake ratio would increase the high kernel elongation characteristic. The heat treatment showed significant difference in all the physicochemical traits of the varieties studied. Heat treatment also affected the very long branch chains of starch, such as amylose. Observation under an electron microscope showed that the samples subjected to heat treatment had more cracks on the tissue structure compared to normal rice samples. The hexagon structure in Mahsuri Mutan produced a greater elongation effect on its kernel. The findings from this study could be useful to breeders in the selection and development of a new high kernel elongation rice variety.

## 1. Introduction

Generally, physicochemical changes are related to the alteration of different rice chemical components which may affect the rice ageing pathway [1,2]. The process of inducing changes in rice in a short time in order to obtain desirable cooking properties, which resemble that of naturally aged rice, is referred to as accelerated or artificial ageing. It can be accomplished by heating rough or milled rice to a high temperature, which will deactivate the enzyme lipase and thus can slow down the rate of lipid oxidation [3,4]. There are three major components that determine the quality of rice: amylose content, kernel elongation and aromatic quality. Cooking quality preferences vary depending on the culture of the country and location [5,6]. Consumers base their concept of quality on the grain’s appearance, i.e., the size and shape of the grain, the behaviour upon cooking, the taste, tenderness, milling quality, appearance and flavour of cooked rice [5].However, in specialty dishes such as Biryani rice, the quality of the cooked rice is important. Most of these dishes have a high kernel elongation rice type, which means the grain will elongate more after cooking and have a better texture than the conventional common rice. Thus, grain size and shape are the first criteria of rice quality that breeders should consider in developing new varieties for commercial production [7,8]. Length/breadth (L/B) filling between 2.5 mm to 3.0 mm and more than 6 mm length is considered widely acceptable [9].

Preferences of grain size and shape differ according to ethnic and geographical locations. Predominantly, long-grain rice is preferred in the Indian sub-continent while Southeast Asia mostly consumes medium–long rice. However, in temperate regions, they prefer short grain varieties. Generally, long-grain rice is mostly demanded in the international market [5,10]. The physical dimensions of rice kernels are an important aspect to determine the quality of grain in the rice industry [11]. The physicochemical characteristics include grain length, breadth, L/B ratio, hulling and milling percentage. The cooking qualities are amylose content (AC), alkali spreading value (ASV), water uptake (WU), volume expansion ratio (VER) and kernel elongation ratio (KER) [12,13]. Grain quality is a very wide area, encompassing diverse characteristics that are directly or indirectly related to the exhibition of one quality type [14,15]. Different cultivars showed significant variations in morphological, physicochemical and cooking properties [16,17]. The cooking quality is an important criterion for the determination of rice grade and market price. Cruz and Khush [5] noted that consumers’ preference is based on the quality of grain, i.e., size and shape, and the quality and taste of the cooked rice.

The genotype (rice variety) and environment (growth conditions) control starch and protein structures (including morphology), and these structures control their properties of interest. Genotype x environment interaction analysis has been reported as a major step that is necessary in developing improved varieties [18,19,20,21]. There are differences in yield and yield component traits, such as starch and protein, when genotypes or varieties are evaluated across environments. Whether the varieties are developed using conventional methods or a marker-assisted approach [22,23,24,25,26,27,28], significant interaction is usually present between the genotype and the environment. A large GxE interaction effect has been reported to affect the progress of selection. Sar et al. [29] studied the effects of variety and growth location on grain composition, and starch structures were determined with the use of three rice varieties which had various compositions of amylose. The three rice varieties were planted at three varied agro-climatic regions of Cambodia. The results indicated an increase in protein and a decrease in lipid contents of polished grains for rice planted in a region with above average temperatures. Starch-fine structures characterized by the chain-length distribution were significantly different among the cultivars [29].

A rice ageing treatment is one of the factors that influences the physical and chemical characteristics of rice. Ageing treatments can improve the cooking quality of rice and increase the kernel elongation rate [30,31]. Due to ageing treatments, significant changes occur in the physicochemical, sensory, cooking and pasting properties of rice [32]. According to Saikrishna et al. [32], aged rice had better commercial value with higher consumer preference in terms of cooked rice texture and flavour. The normal ageing takes more than six months to achieve good quality. However, Normand [33] recommended an artificial ageing for the rice grain, where the rice grain was heated in 90 °C for 3 h in a closed container, which mimics the normal ageing treatment. Abdullah et al. [34] reported a significant change in tissue structure of the Mahsuri, Mahsuri Mutant and Puteri varieties under a light microscope after exposure to the ageing treatment. The present study was conducted in order to determine the ageing effects on the physicochemical properties of three rice varieties, namely Mahsuri Mutant, MR219 and Basmati 370, and to compare the effects of the ageing treatment on tissue structure between Mahsuri and Mahsuri Mutant.

## 2. Materials and Methods

### 2.1. Source Population

Two high kernel elongation rice varieties, Mahsuri Mutant and Basmati 370, were collected from Malaysia and India, respectively. Another variety (MR219) with characteristic normal kernel elongation was collected from the Malaysian Agricultural Research and Development Institute (MARDI). The three varieties were utilized in a breeding programme to develop a new variety of rice with characteristic high kernel elongation, as well as high yielding. Mahsuri Mutant and Basmati 370 served as the donor parents with a genetic background of high kernel elongation. The variety MR219 served as a recipient parent with characteristic high yielding but not high elongation. The parental varieties were utilized in a backcross breeding programme for varietal development.

### 2.2. Artificial Ageing Treatment

The samples were heated at 90 °C, in an oven (Memmert, Schwabach, Germany), for 3 h. After the heat treatment, the samples were cooled at room temperature (25 °C) for 1 h according to the method described by Faruq et al. [35]. Then, the samples were cooked in order to determine the kernel elongation ratio.

### 2.3. Determination of Physicochemical Properties

#### 2.3.1. Alkali Digestion Value

Ten milled kernels were placed in 10 mL of 1.7% potassium hydroxide (KOH) solution in a petri dish, and the grains were arranged in a manner that they did not touch each other. The grain samples were kept for 23 h at 30 °C and the data on score spreading were recorded following the alkali spread value described by Oko et al. [36].

#### 2.3.2. Water Uptake Ratio (WUR)

This was determined by cooking 2.0 g of whole rice kernels from each treatment in 20 mL distilled water for 20 min in a boiling water bath [36]. After that, excess water was drained out from the cooked rice. The cooked rice samples were weighed, and the water uptake ratio was calculated as follows:

$$\begin{array}{c} Water\;uptake\;ratio\;=\;\underline{Weight\;of\;cooked\;rice}\\ \;\;\;\;\;\;\;\;\;\;Weigh\;of\;uncooked\;rice\;sample \end{array}$$

#### 2.3.3. Solids in Cooking Water (SCW)

The method modified by Oko et al. [36] was used to determine the drying aliquot (excess cooked rice water). The empty Petri dish was weighed (*W*_1_), then aliquot samples were put into the Petri dish and weighed (*W*_2_).Next, the Petri dish with the aliquot was put in the oven at 40 °C for 1 h to let the water evaporate. Finally, the weight of the Petri dish with dried aliquot was recorded (*W*_3_). The solid content was determined with the formula:

$$Solids\;in\;cooking=\;\;W_{3}\;-\;W_{1}$$

#### 2.3.4. High Kernel Elongation (HKE) Ratio

The average lengths of ten grains of milled rice of six generations in three replications were used for HKE. The rice kernel was soaked in tap water for 20 min in a 20 mL test tube. Then, the test tubes containing the samples were put in boiling water for 30 min. The water in the test tube was drained out and the cooked rice was placed on a glass sheet for a few minutes to evaporate the excess moisture, and then we measured the length of the cooked rice. The measurement was performed using a digital Vernier calliper. The elongation ratio was measured according to the method described by Golam et al. [37].

$$\begin{array}{l} Elongation\;ratio\;=\;\underline{Average\;of\;cooked\;\mathrm{kernels}}\\ \;\;\;\;\;\;\;\;\;\;\;\;\;\;\;\;\;\;\;\;\;\;Average\;length\;of\;raw\;\;\mathrm{kernels} \end{array}$$

### 2.4. Amylose Content

A sample of ground rice (0.10 g) was put into a 100 mL volumetric flask and 95% (*v*/*v*) 1 mL ethanol was added. The mixture was slightly shaken to ensure the entire sample was wet. Then, 9 mL of 1M sodium hydroxide (NaOH) was added and mixed thoroughly. After that, the volumetric flask was heated in a hot water bath to dissolve the starch. Then, the mixture was cooled to room temperature before the addition of distilled water. The amylose assay was prepared by adding 0.1 mL (*v*/*v*) acetic acid to 5mL of distilled water, then a 0.5 mL aliquot and 0.2 mL iodine solution was added before the addition of distilled water for final volume (10 mL). Then, the assay was mixed using a Vortex mixer (VelpScientifica, Schwerte, Germany). The absorbance was measured at 720 nm against a blank solution by using a spectrophotometer (Varian, Palo Alto, CA, USA). A calibration curve was plotted against the three tested varieties [38]. The samples were categorized as waxy, very low amylose, low, intermediate and high given 1–2, 2–9, 10–20, 20–25 and 25–30 percent amylose contents, respectively. The starch structure of milled rice (ageing and non-ageing) and cooked rice (ageing and non-ageing) of Mahsuri and Mahsuri Mutant were observed under a Scanning Electron Microscope (SEM) at the Institute Bioscience, Universiti Putra Malaysia (UPM). The samples were viewed at 2000× magnification.

### 2.5. Determination of Starch Chain-Length Distribution

The starch contents in MR219, Mahsuri Mutant and B370 were isolated in the ITAFoS UPM laboratory following the procedure described by Lim et al. [39]. The modified Schoch procedure according to Jane and Chen [40] was adopted in separating amylose and amylopectin contents, including the intermediate components. A mixture of amylopectin and its intermediates was purified by five times recrystallization in order to exclude amylose remnants. The gel-permeation chromatography (GPC) was used to examine the purity of amylopectin observed by the total carbohydrate (CHO) and blue value. An automatic Potentiometric Titrator was used to measure the iodine affinities of defatted starch and that of isolated amylopectin. This followed the procedure of Kasemsuwan et al. [41]. Apparent and Absolute amylose contents were calculated following the methods described by Takeda and Hizukuri [42] and Kasemsuwan et al. [41], respectively. Debranching of amylopectin by isoamylase followed the method reported by Jane and Chen [40]. A high-performance anion-exchange chromatography system [40] was used to analyse the amylopectin branch chain length distributions.

### 2.6. Statistical Analysis

Data collected on physicochemical properties and ageing vs. non-ageing treatments were subjected to analysis of variance (ANOVA) using SAS software version 9.4 [43]. Additionally, descriptive statistics, such as mean, range, standard deviation and coefficient of variation (CV), were calculated for each trait. Mean comparisons were performed using a least significant difference (LSD) test at a 95% and 99% confidence level. Correlation coefficients were also analysed using SAS Software programme version 9.4 to study the relationship between traits [43,44,45].

## 3. Results

### 3.1. Physicochemical Properties of High Kernel Elongation Rice

The physicochemical characteristics, such as water uptake ratio, alkali spread value (%), kernel elongation ratio and amylose content of the grain among the three sourced varieties with ageing treatment, are as presented in Table 1. Highly significant differences were found among the three varieties and between ageing treatments on all the physicochemical characteristics. Basmati 370 had the highest solid cooking (quantity of water leached during cooking) percentage while MR219 had the lowest value, which was not significantly different from the solid cooking percentage recorded in Mahsuri Mutant. For the kernel elongation ratio, Basmati 370 had the highest value of 2.71, followed by Mahsuri Mutant, whereas MR219 had the lowest value of 1.14. Contrarily, Mahsuri Mutant had the highest value of 4.1, which is significantly higher than MR219 with 4.35. However, the lowest value of alkaline spreading was observed in Basmati 370. For amylose content, the highest value was observed in MR219 followed by Mahsuri Mutant and Basmati 370 at 24.63, 24.03 and 20.3, respectively. The result in Table 2 shows interaction between variety and ageing treatments on solid content, alkali spreading value and water uptake ratio

#### 3.1.1. Correlation among Physicochemical Characteristics

The correlation coefficients among the physicochemical traits are presented in Table 3. High kernel elongation had a significant positive correlation with the water uptake ratio and also positively correlated with solid content. However, HKE had non-significant correlation with amylose content and alkali spreading value (gelatinization). This finding showed that the water uptake ratio during rice cooking was directly affected by the quality of rice elongation. The results showed that amylose content had a negative correlation with WUR but positively correlated with ASV.

#### 3.1.2. Absolute Amylose Content

The result in Table 4 shows both the apparent and absolute amylose contents. The long branch chains of starch, such as amylose and amylopectin, are known to bind iodine and form a single helical complex during potentiometric titration. They develop a blue colour and thereafter inflate the iodine affinity and the apparent amylose starch content. The normal starches showed that all the rice varieties had higher apparent amylose contents compared to their absolute amylose contents. Iodine affinities of waxy starches, very low, low, intermediate and high amylose are presented in Table 5. The HPAEC-ENZ-PAD chromatography results obtained (Table 6) indicated that amylopectin that did not have detectable very long branch chains (dp above 73) showed no detectable iodine affinity. This result is an indication of positive association between the presence of very long branch chains of amylopectin and the iodine affinity of amylopectin against long–average branch chain lengths (BCL). The BCL distribution of amylopectin normalized HPAEC-ENZ-PAD chromatograms of BCL distributions of amylopectins isolated from the starches, which displayed A-type X-ray patterns, and the results are presented in Table 6. The A-type starches had higher proportions of short chains and lower proportions of long chains (Table 6).

#### 3.1.3. Examination of Kernel Structure through Electron Microscope (SEM)

**Figure 1:**
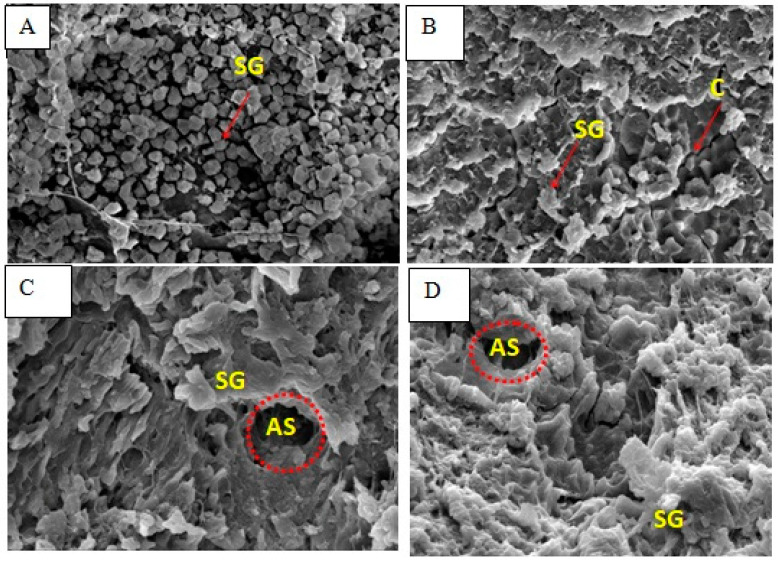
Endosperm morphology of Mahsuri variety. Non-ageing milled rice (**A**), ageing milled rice (**B**), non-ageing cooked rice (**C**) and ageing cooked rice (**D**). C = Cracking between starch granule; SG = Starch granule; AS = Air space between structure.

**Figure 2:**
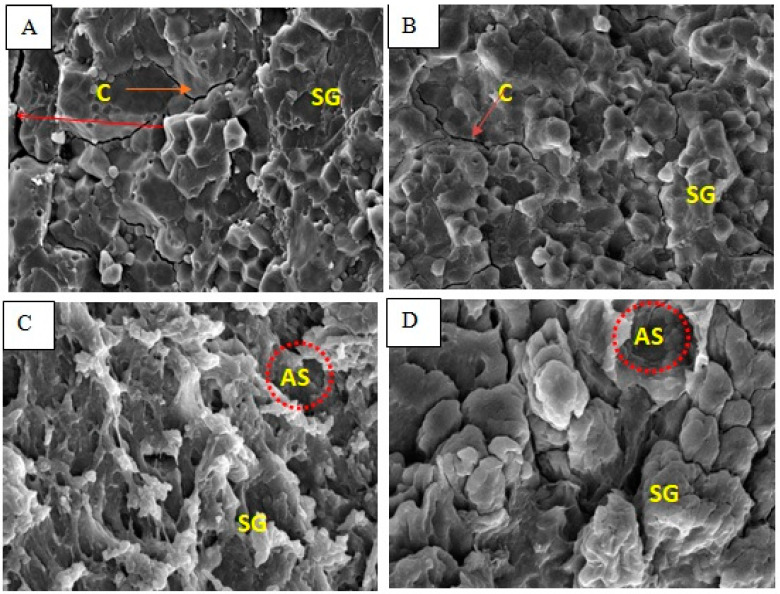
Endosperm morphology of Mahsuri Mutant variety. Non-ageing milled rice (**A**), ageing milled rice (**B**), non-ageing cooked rice (**C**) and ageing cooked rice (**D**). C = Cracking between starch granule; SG = Starch granule; AS = Air space between structure.

The endosperm appearance of non-ageing milled and cooked rice of Mahsuri (Figure 1) was examined in comparison with non-ageing milled and cooked rice of Mahsuri Mutant (Figure 2). The endosperm of non-ageing milled Mahsuri is packed and granule. Meanwhile, the ageing Mahsuri is loosely packed and showed some cracking between the starch granules. The non-ageing starch granules are more compact than the ageing milled rice of Mahsuri. The ageing effect caused the structure to form a crack between the starch granules because of hydration and lipid oxidation that occurred during the ageing process. In addition to the observations from SEM results on ageing and non-ageing milled rice, there were also different structural observations due to the physicochemical changes associated with the ageing and non-ageing of cooked rice. The endosperm of elongating and non-elongating varieties showed differences in the shape and arrangement of cells. The structural arrangement of starch molecules within cells might influence the elongation pattern where the long belt or radially arranged cells in the non-elongating rice might restrict linear expansion of cells and allow the intermittent area to expand outwardly, resulting in the breadthwise swelling.

## 4. Discussion

In the ageing treatment, a higher value was observed for the entire number of traits in the ageing treatment as compared with the non-ageing. This result showed that Basmati 370 structure was softer and easier to reach out based on solid cooking parameters. Shamin et al. [46] reported a higher cooking solid value in Basmati with a shorter cooking time. The presence of the interaction between varieties and the ageing treatment for solid cooking, alkaline spreading value and water uptake ratio were presented in Table 1 and Table 2. Based on the results, there was significant difference between the ageing and non-ageing solid content of all the varieties except Basmati 370, whereas for alkali spreading value and water uptake ratio, all the varieties had significance differences between ageing and non-ageing treatments. Saikrishna et al. [32] reported that storage or ageing treatment increases chemical reaction activity during storage. Based on the study, amylose content was significantly affected by the ageing period.

The results obtained on the correlation of physicochemical traits were in contrast with the findings made by Saikrishna et al. [32], where the increase in water uptake value simultaneously increased the amylose content in cooked rice. Although, the ageing treatment may influence the results where the water uptake ratio was higher in ageing rice due to physical properties of the rice. After ageing, some cracking developed on the rice structure due to a decrease in moisture content. This result had a direct effect on the parameters: water uptake ratio and solid cooking leached upon the cooking period. Aged, milled rice has greater volume expansion and water absorption and less dissolved solid in cooking [47]. In this study, the grain elongation was positively correlated with solid content during cooking. However, Prodhan et al. [47] mentioned in his study that the overall changes may depend on the rice variety, storage condition and further treatment. He also mentioned that the ageing process affected eating quality. Only the water uptake ratio and solid content after cooking showed positive and highly significant correlation with high kernel elongation at r = 0.019 and r = 0.829, respectively. It was confirmed in Binoth et al. [48] that there was significant and positive correlation between the water uptake ratio and the expanded ratio which affected the kernel elongation. The same results were reported by Tomar and Nanda [49] and Deosarkar and Nerkar [50]. The non-significant correlation coefficient obtained in amylose content and high kernel elongation was in contrast with the findings made by Wu et al. [51], who reported that elongation ratio showed highly significant positive association with amylose content. However, rice variety could also influence the results. In the development of improved breeding lines of superior quality, the correlation among grain quality traits is useful in the choice of parents, as well as in the screening and selection procedures for the segregating populations.

The results obtained from this study on absolute amylose content correspond with the findings of other researchers such as Hanashiro et al. [52], Cameron and Donald [53] and Jane et al. [54]. More detailed results were obtained for long branch chain length distribution of amylopectin with the aid of the HPAEC-ENZ-PAD chromatograms used. Some of the samples of starch analysed shouldered at dp 18–21. A similar result was reported in the work of Hanashiro et al. [52]. The higher hardness and lower adhesiveness are likely to be associated with a lower hydration process of starch granules in aged rice grains stored at a higher temperature [55].According to Faruq et al. [30], the internal anatomical structure of the rice kernel, cell shape, and their arrangement might influence the water uptake and nature of the swelling of the kernel during cooking. Therefore, the internal structure of rice grains could represent the effects of ageing for cooking evaluation. In the previous study of Faruq et al. [30], they observed that the internal cracks (vacuum-like structure) observed in aged rice kernels were higher than in non-aged kernels. The same situation also happens in the non-ageing and ageing of milled rice of the Mahsuri Mutant variety. However, the variety could also influence the amount of cracking after the ageing treatment [30].With the comparison between Mahsuri as a wild-type variety and Mahsuri Mutant, which is modified variety from Mahsuri, the cracking number is higherin Mahsuri Mutant. These observations indicated that the internal structure of Mahsuri Mutant is loose compared to Mahsuri. Moreover, from the observation, the shape of the internal structure between Mahsuri and Mahsuri Mutant were also quite different, where the internal shape of Mahsuri Mutant is most likely a round shape compared to Mahsuri which has more of a flaky shape. The shape of Mahsuri Mutant is similar to that of Basmati rice: a high kernel elongation type of rice [56]. Hormdok and Noomhorm [57] reported that the physicochemical properties changed during the ageing process in rice.

According to Kanlayakrit and Maweang [58], the elongating rice cells of uniform size and shape might influence lengthwise swelling. Following the study on the Basmati variety’s rice structure, which was categorized as a high kernel elongation variety, the tissue structure had nearly equidistant pentagonal or hexagonal cells arranged in a honeycomb pattern. This is different from the non-elongating variety, where the cells were arranged in a rectangular shape and radially in a column extending from the centre to the periphery in breadthwise swelling-type after cooked. Additionally, there is the probability that a lipoprotein matrix at the sub-cellular level might influence the gelatinization and swelling of starch molecules in a specific direction. The internal structure of cooking rice obviously changes after cooking for both varieties. The previous shape changes due to the physical and chemical changes during cooking activities. The shape looks flaky and more air space is formed. This happens before and after ageing treatment. However, a different situation happens in Mahsuri Mutant where, after ageing, the internal granules were round shaped and arranged. Under the normal rice cooking condition, the SEM displayed uneven structure covered by the formed films.

Artificial ageing involved heat treatment on milled rice and caused the cracking structure to form between the starch granules. This could be a result of hydration during the heat treatment. So, the hydration of the rice kernels was increased with temperature rise, which facilitated the leaching of carbohydrates. From the histological study of artificially ageing rice (heat treatment on rice grain) that was performed by Tamura et al. [59], as the temperature increased, there was more cracking on the rice structure. The lines on the structure were observed between the ventral and dorsal sides. As the temperature increased ≥80°C, the cracks spread and some became voids, and the cooked grain was swelling as the cracking fissure increased. Of great interest was the longitudinal grain swelling which contributed to more kernel elongation when cooked. This study showed that artificial ageing could give the same results as traditional ageing (storing for 3 to 4 month) as reported by Golam et al. [37].

The appearance of the hollows can be explained by the fact that, during cooking, the starch located at the surface could leach out easily into the water, resulting in high void density at the exterior surface [60,61]. Additionally, the internally stored starch absorbs water through voids and swells greatly, disrupting the cell walls beneath the surface [59]. The situation could affect the cell wall after the ageing process. The morphological properties of cooked rice correspond to the texture properties. After the AM and AP diffused out of the swollen granules and leached into the cooking water, the starch granules were disrupted and lost rigidity. The framework of the rice grains lost its support, and the structure of the rice grains became very loose, which resulted in the formation of a fluffy, soft and non-sticky texture of cooked rice [61]. The packed arrangement of the starch granules shows the effect of the expansion of the starch granules during cooking. The air space produces a lengthwise effect during the cooking process where the expansion focused on the straight direction and less on breadthwise expansion. Meanwhile, with normal rice, the starch granule expansion during cooking focuses on breadthwise expansion and more air space between the starch granules. These actions affect the cooked rice’s physical properties and the texture of the cooked rice. The lengthwise expansion produces a fluffiness effect, just like the ageing effect. Ageing causes the physical and internal structure of the rice to change, leading to expansion of the rice. Furthermore, the strength of the wall structure produced an impact on the elongation of the rice. According to Chandi and Sogi [62], high elongation and low solid loss in Pusa Basmati 1 rice may be attributed to the greater strength of the cell wall line, which is able to hold the pressure until maximum elongation takes place without rupturing of the cell wall. This is why Mahsuri Mutant could elongate better than Mahsuri.

## 5. Conclusions

Based on the SEM observation, there was more cracking on the ageing tissue structure surface. This cracking can affect the ability of the kernel to absorb water and to elongate during the cooking period. The reason for the cracking phenomenon in an ageing kernel is due to a decrease in moisture content. Therefore, more space (vacuum) was created for the kernel to absorb more water and elongate. There was more cracking on aged Mahsuri Mutant compared to Mahsuri. This is one of the reasons a Mahsuri Mutant’s kernels can elongate better than a Mahsuri’s kernel. Furthermore, the shape of the tissue structure was a main factor in the characteristic changes, where in Mahsuri Mutant, the starch granule shape was similar to that of Basmati 370. Ageing treatment had a positive influence on major cooking quality traits, such as kernel elongation, water absorption, alkali spreading value and water uptake ratio. Additionally, this ageing can improve the quality of rice and its marketability could be widened. The endosperm of non-ageing milled Mahsuri rice is tightly packed and granulated. Meanwhile, the ageing Mahsuri is loosely packed and had some cracking between the starch granules. The non-ageing starch granules were more compact than the ageing milled rice of Mahsuri. The ageing effect causes the structure to form a crack between the starch granules because of hydration and lipid oxidation which occurred during the ageing process. The changes in the internal structure of rice could induce the water absorption, and the lengthwise or breadthwise effect could happen during cooking based on the rice structure of that variety. Just like amylose, the very long branch chain length of amylopectin has the potential to bind iodine and increase the iodine affinity. Ageing treatment also affected the very long branch chain of starch, such as amylose. From this study, it was therefore concluded that ageing (heat) treatment has a direct effect on increasing HKE in rice populations and could also amplify the expression of grain kernel characteristics. The current findings could be useful to breeders for specialty rice varietal development in the future.

## Figures and Tables

**Table 1:** Analysis of variance (ANOVA) showing main and interaction effects among rice varieties and ageing effects on physicochemical properties.

Factor	SC (Leached) (g)	HKE	ASV	AC (mg/L)	WUR
**Rice varieties** **(RV)**					
MR219	0.07 b	1.44 c	4.35 b	24.63 a	9.35 b
Mahsuri Mutant	0.08 b	2.15 b	5.10 a	24.03 b	7.45 c
B370	0.17 a	2.71 a	3.30 c	20.03 c	10.7 a
**LSD Value**	**0.0046**	**0.1065**	**0.07**	**0.5928**	**0.0719**
**Ageing treatment** **(AT)**					
Ageing	0.153 a	2.425 a	4.575 a	24.26 a	8.46 a
Non-Aging	0.116 b	2.131 b	3.83 b	22.74 b	9.29 b
**LSD value**	**0.0038**	**0.087**	**0.0572**	**0.484**	**0.0587**
**RV**	0.021 **	3.266 **	6.540 **	50.148 **	**21.311 ns**
**AT**	0.007 **	0.481 **	2.006 **	13.009 **	**27.468 ns**
**RV × AT**	**	**ns**	**	**ns**	**

**Note:** Means within a factor and column followed by the same letter are not significantly different at *p* ≤ 0.05 by using LSD test for ANOVA; ** = Highly significant at *p* ≤ 0.01; ns = Not significant at *p* = 0.05; SC = Solid content; HKE = High kernel elongation; ASV = Alkali spread value and WUR = Water uptake ratio, RV = Rice variety, AT = Ageing treatment.

**Table 2:** Interaction between variety and ageing treatments on solid content, alkali spreading value and water uptake ratio.

Factor	SC (Leached) (g)		ASV		WUR	
Rice Varieties (RV)	A	NA	A	NA	A	NA
MR219	0.069 e	0.0825 c	4.45 c	4.25 e	11.242 e	7.4600 b
Mahsuri Mutant	0.138 b	0.02175 d	5.4 a	4.8 b	8.603 d	6.3000 f
B370	0.1675 a	0.16675 a	4.35 d	2.25 f	12.163 a	9.2398 c

**Note:** Means within a factor and column followed by the same letter are not significantly different at *p* ≤ 0.05 by using LSD test for ANOVA. SC = Solid content; ASV = Alkali spread value and WUR = Water uptake ratio.

**Table 3:** Correlation coefficients (r) of physicochemical properties of the rice varieties studied.

Physiochemical Characteristics	WUR	SC	HKE	ASV	AC
**WUR**	1.00	0.107 ns	0.019 *	−0.153 ns	−0.427 **
**SC**		1.00	0.829 **	−0.353 *	0.026 ns
**HKE**			1.00	−0.264 ns	−0.206 ns
**ASV**				1.00	0.626 **
**AC**					1.00

**Note:** * significant at 0.05 probability level, ** highly significant at 0.01 probability level, WUR: Water uptake ratio, SC: Solid content, HKE: High Kernel elongation, ASV: Alkali spread value, AC: Amylose content.

**Table 4:** Iodine affinities and percent amylose contents of starch.

	Iodine Affinity		Percent Amylose Content		
Rice Variety/Source	Starch	Amylopectin	Apparent (A)	Absolute (B)	D = A − B
A-type starch					
MR219	5.44 ± 0.16	1.34 ± 0.02	27.6	24.63	2.97
Mahsuri mutant	5.66 ± 0.13	0.65 ± 0.06	25.2	24.03	1.17
Basmati 370	5.10 ± 0.01	1.12 ± 0.01	24.98	20.03	4.95

**Table 5:** Iodine affinities and amylose contents of waxy starch and amylose.

Source	Iodine Affinity	Apparent Amylose Content (%)
A-type starch		
Waxy starch	0.001 ± 0.0	0.0
Very low	0.43 ± 0.02	1.4
Low	0.01 ± 0.01	2.0
Intermediate	0.42 ± 0.02	2.3
High	0.76 ± 0.02	3.5

**Table 6:** Branch chain length (BCL) distribution of amylopectin.

	Peak dp		Average	Distribution (%)					Maximum Detectable dp
Rice Type/Source	I	II	CL	dp 6–9	dp 6–12	dp 13–24	dp 25–36	dp > 37	
A-type starch									
MR219	12	47	23.6	4.22	20.5	53.4	13.4	17.6	81
Mahsuri mutant	12	46	22.5	8.69	24.6	49.65	14.6	15	79
Basmati 370	12	42	19.8	9.66	28.5	54.33	13.6	7.7	67
Ageing rice	13	48	24.2	3.87	17.8	46.8	14.7	18.9	82
Non-ageing rice	12	43	22.3	4.9	20.6	47.8	16.9	13.2	77

## Data Availability

The data presented in this study are available on request from the corresponding author.

## References

[1] Le T.Q., Songsermpong S., Rumpagaporn P., Suwanagul A., Wallapa S. (2014). Microwave heating for accelerated aging of paddy and white rice. Aust. J. Crop Sci..

[2] Ab. Halim A.A., Rafii M.Y., Osman M.B., Oladosu Y., Chukwu S.C. (2021). Ageing effects, generation means, and path coefficient analyses on high kernel elongation in mahsurimutan and basmati 370 rice populations. BioMed Res. Int..

[3] Jaisut D., Prachayawarakorn S., Varanyanond W., Tungtrakul P., Soponronnarit S. (2009). Accelerated aging of jasmine brown rice by high-temperature fluidization technique. Food Res. Int..

[4] Ahmed M.A., Rafii M.Y., Izzati M.N., Khalilah A.K., Awad E.A., Kaka U., Chukwu S.C., Liang J.B., Sazili A.Q. (2022). Biological additives improved qualities, in vitro gas production kinetics, digestibility, and rumen fermentation characteristics of different varieties of rice straw silage. Anim. Prod. Sci..

[5] Cruz N.D., Khush G.S. (2000). Rice grain quality evaluation procedures. Aromat. Rices.

[6] Sarif H.M., Rafii M.Y., Ramli A., Oladosu Y., Musa H.M., Rahim H.A., Zuki Z.M., Chukwu S.C. (2020). Genetic diversity and variability among pigmented rice germplasm using molecular marker and morphological traits. Biotechnol. Biotechnol. Equip..

[7] Rani N.S., Pandey M.K., Prasad G.S., Sudharshan I. (2006). Historical significance, grain quality features and precision breeding for improvement of export quality basmati varieties in India. Indian J. Crop Sci..

[8] Chukwu S.C., Rafii M.Y., Oladosu Y., Okporie E.O., Akos I.S., Musa I., Swaray S., Jalloh M., Al-Mamun M. (2022). Genotypic and phenotypic selection of newly improved putra rice and the correlations among quantitative traits. Diversity.

[9] Sujatha S.J., Ahmad R., Bhat P.R. (2004). Physicochemical properties and cooking qualities of two varieties of raw and parboiled rice cultivated in the coastal region of Dakshina Kannada, India. Food Chem..

[10] Oladosu Y., Rafii M.Y., Magaji U., Abdullah N., Miah G., Chukwu S.C., Hussin G., Ramli A., Kareem I. (2018). Genotypic and phenotypic relationship among yield components in rice under tropical conditions. BioMed Res. Int..

[11] Fitzgerald M.A., McCouch S.R., Hall R.D. (2009). Not just a grain of rice: The quest for quality. Trends Plant Sci..

[12] Verma D.K., Mohan M., Prabhakar P.K., Srivastav P.P. (2015). Physico-chemical and cooking characteristics of Azad basmati. Int. Food Res. J..

[13] Okporie E.O., Chukwu S.C., Onyishi G.C., Ekwu L.G., Oko G.O. (2014). Increase in protein, oil, amylose and amylopectin contents of two populations of maize (*Zea mays* L.) after two cycles of reciprocal recurrent selection. IOSR J. Agric. Vet Sci..

[14] Siddiqui S.U., Kumamaru T., Satoh H. (2007). Pakistan rice genetic resources-I: Grain morphological diversity and its distribution. Pak. J. Bot..

[15] Okporie E.O., Chukwu S.C., Onyishi G.C. (2013). Phenotypic recurrent selection for increase yield and chemical constituents of maize (*Zea mays* L.). World Appl. Sci. J..

[16] Yadav R.B., Khatkar B.S., Yadav B.S. (2007). Morphological, physicochemical and cooking properties of some Indian rice (*Oryza sativa* L.) cultivars. J. Agric. Technol..

[17] Sabri R.S., Rafii M.Y., Ismail M.R., Yusuff O., Chukwu S.C., Hasan N.A. (2020). Assessment of agro-morphologic performance, genetic parameters and clustering pattern of newly developed blast resistant rice lines tested in four environments. Agronomy.

[18] Ebem E.C., Afuape S.O., Chukwu S.C., Ubi B.E. (2021). Genotype× environment interaction and stability analysis for root yield in sweet potato [*Ipomoea batatas* (L.) lam]. Front. Agron..

[19] Salleh S.B., Rafii M.Y., Ismail M.R., Ramli A., Chukwu S.C., Yusuff O., Hasan N.A. (2022). Genotype-by-environment interaction effects on blast disease severity and genetic diversity of advanced blast-resistant rice lines based on quantitative traits. Front. Agron..

[20] Akos I.S., Yusop M.R., Ismail M.R., Ramlee S.I., Shamsudin N.A., Ramli A.B., Haliru B.S., Ismai’la M., Chukwu S.C. (2019). A review on gene pyramiding of agronomic, biotic and abiotic traits in rice variety development. Int. J. Appl. Biol..

[21] Akos I.S., Rafii M.Y., Ismail M.R., Ramlee S.I., Shamsudin N.A., Ramli A., Chukwu S.C., Swaray S., Jalloh M. (2021). Evaluation of inherited resistance genes of bacterial leaf blight, blast and drought tolerance in improved rice lines. Rice Sci..

[22] Oladosu Y., Rafii M.Y., Chukwu S.C., Fatai A., Magaji U., Kareem I., Kamarudin Z.S., Muhammad I.I., Kolapo K. (2019). Drought resistance in rice from conventional to molecular breeding: A review. Int. J. Mol. Sci..

[23] Chukwu S.C., Rafii M.Y., Ramlee S.I., Ismail S.I., Hasan M.M., Oladosu Y.A., Magaji U.G., Akos I., Olalekan K.K. (2019). Bacterial leaf blight resistance in rice: A review of conventional breeding to molecular approach. Mol. Biol. Rep..

[24] Chukwu S.C., Rafii M.Y., Ramlee S.I., Ismail S.I., Oladosu Y., Okporie E., Onyishi G., Utobo E., Ekwu L., Swaray S. (2019). Marker-assisted selection and gene pyramiding for resistance to bacterial leaf blight disease of rice (*Oryza sativa* L.). Biotechnol. Biotechnol. Equip..

[25] Oladosu Y., Rafii M.Y., Arolu F., Chukwu S.C., Muhammad I., Kareem I., Salisu M.A., Arolu I.W. (2020). Submergence tolerance in rice: Review of mechanism, breeding and, future prospects. Sustainability.

[26] Chukwu S.C., Rafii M.Y., Ramlee S.I., Ismail S.I., Oladosu Y., Kolapo K., Musa I., Halidu J., Muhammad I.I., Ahmed M. (2019). Marker-assisted introgression of multiple resistance genes confers broad spectrum resistance against bacterial leaf blight and blast diseases in Putra-1 rice variety. Agronomy.

[27] Chukwu S.C., Rafii M.Y., Ramlee S.I., Ismail S.I., Oladosu Y., Muhammad I.I., Musa I., Ahmed M., Jatto M.I., Yusuf B.R. (2020). Recovery of recurrent parent genome in a marker-assisted backcrossing against rice blast and blight infections using functional markers and SSRs. Plants.

[28] Chukwu S.C., Rafii M.Y., Ramlee S.I., Ismail S.I., Oladosu Y., Muhammad I.I., Ubi B.E., Nwokwu G. (2020). Genetic analysis of microsatellites associated with resistance against bacterial leaf blight and blast diseases of rice (*Oryza sativa* L.). Biotechnol. Biotechnol. Equip..

[29] Seila S.A., Tizzotti M.J., Hasjim J., Gilbert R.G. (2014). Effects of rice variety and growth location in Cambodia on grain composition and starch structure. Rice Sci..

[30] Faruq G., Prodhan Z.H., Nezhadahmadi A. (2015). Effects of ageing on selected cooking quality parameters of rice. Int. J. Food Prop..

[31] Oladosu Y., Rafii M.Y., Arolu F., Chukwu S.C., Salisu M.A., Fagbohun I.K., Muftaudeen T.K., Swaray S., Haliru B.S. (2022). Superabsorbent polymer hydrogels for sustainable agriculture: A review. Horticulturae.

[32] Saikrishna A., Dutta S., Subramanian V., Moses J.A., Anandharamakrishnan C. (2018). Ageing of rice: A review. J. Cereal Sci..

[33] Normand F.L. (1966). Process of Ageing Rice Artificially. U.S. Patent.

[34] Abdullah N.S., Abdullah M.Y., Ghaffar M.B., Awal A., Aziz N.A., Abdullah S. (2018). Traits Performance and Heterosis Estimation in F1 Rice Generations Crossed between Basmati 370 and Selected Malaysian Rice Varieties. Pertanika J. Trop. Agric. Sci..

[35] Faruq G., Mohamad O., Hadzim M., Meisner C.A., Perai S. (2003). Optimization of aging time and temperature for four Malaysian rice cultivars. Pak. J. Nutr..

[36] Oko A.O., Ubi B.E., Dambaba N. (2012). Rice cooking quality and physico-chemical characteristics: A comparative analysis of selected local and newly introduced rice varieties in Ebonyi State, Nigeria. Food Public Health.

[37] Faruq G., Khalid N., Jennifer A.H., Subha B., Zulqarnain M., Osman M., Nazia A.M., Mohammad O. (2010). Evaluation of kernel elongation ratio and aroma association in global popular aromatic rice cultivars in tropical environment. Afr. J. Agric. Res..

[38] Fitzgerald M.A., Bergman C.J., Resurreccion A.P., Möller J., Jimenez R., Reinke R.F., Martin M., Blanco P., Molina F., Chen M.H. (2009). Addressing the dilemmas of measuring amylose in rice. Cereal Chem..

[39] Lim S.T., Kasemsuwan T., Jane J. (1994). Characterization of phosphorus in starches using 31P-NMR spectroscopy. Cereal Chem..

[40] Jane J.L., Chen J.F. (1992). Effect of amylose molecular size and amylopectin branch chain length on paste properties of starch. Cereal Chem..

[41] Kasemsuwan T., Jane J., Schnable P., Stinard P., Robertson D. (1995). Characterization of the dominant mutant amylose-extender (Ae1-5180) maize starch. Cereal Chem..

[42] Takeda Y., Hizukuri S., Juliano B.O. (1987). Structures of rice amylopectins with low and high affinities for iodine. Carbohydr. Res..

[43] SAS (2013). The SAS System for Windows.

[44] Chukwu S.C., Ekwu L.G., Onyishi G.C., Okporie E.O., Obi I.U. (2013). Correlation between agronomic and chemical characteristics of maize (*Zea mays* L.) genotypes after two years of mass selection. Int. J. Sci. Res..

[45] Hashim N., Rafii M.Y., Oladosu Y., Ismail M.R., Ramli A., Arolu F., Chukwu S. (2021). Integrating multivariate and univariate statistical models to investigate genotype–environment interaction of advanced fragrant rice genotypes under rainfed condition. Sustainability.

[46] Shamim F., Raza M.A., Akhtar M. (2017). Grain quality attributes of new Rice Basmati lines of Pakistan. J. Appl. Agric. Biotechnol..

[47] Prodhan Z.H., Faruq G., Taha R.M., Rashid K.A. (2017). Agronomic, transcriptomic and metabolomic expression analysis of aroma gene (badh2) under different temperature regimes in rice. Int. J. Agric. Biol..

[48] Binodh A.K., Kalaiyarasi R., Thiyagarajan K. (2010). Genetic divergence of rice varieties and hybrids for quality traits. ORYZA-Int. J. Rice.

[49] Tomar J.B., Nanda J.S. (1982). Inheritance of cooking quality components in rice. Oryza.

[50] Deosarkar D.B., Nerkar Y.S. (1994). Correlation and path analysis for grain quality characters in indica rice. J. Maharashtra Agric. Univ..

[51] Wu L., Zhang W., Ding Y., Zhang J., Cambula E.D., Weng F., Liu Z., Ding C., Tang S., Chen L. (2017). Shading contributes to the reduction of stem mechanical strength by decreasing cell wall synthesis in japonica rice (*Oryza sativa* L.). Front. Plant Sci..

[52] Hanashiro I., Abe J.I., Hizukuri S. (1996). A periodic distribution of the chain length of amylopectin as revealed by high-performance anion-exchange chromatography. Carbohydr. Res..

[53] Cameron R.E., Donald A.M. (1992). A small-angle X-ray scattering study of the annealing and gelatinization of starch. Polymer.

[54] Jane J.L., Chen Y.Y., Lee L.F., McPherson A.E., Wong K.S., Radosavljevic M., Kasemsuwan T. (1999). Effects of amylopectin branch chain length and amylose content on the gelatinization and pasting properties of starch. Cereal Chem..

[55] Keawpeng I., Venkatachalam K. (2015). Effect of aging on changes in rice physical qualities. Int. Food Res. J..

[56] Golam F., Prodhan Z.H. (2012). Kernel elongation in rice. J. Sci. Food Agric..

[57] Hormdok R., Noomhorm A. (2007). Hydrothermal treatments of rice starch for improvement of rice noodle quality. LWT-Food Sci. Technol..

[58] Kanlayakrit W., Maweang M. (2013). Postharvest of paddy and milled rice affected physicochemical properties using different storage conditions. Int. Food Res. J..

[59] Tamura M., Nagai T., Hidaka Y., Noda T., Yokoe M., Ogawa Y. (2014). Changes in histological tissue structure and textural characteristics of rice grain during cooking process. Food Struct..

[60] Rewthong O., Soponronnarit S., Taechapairoj C., Tungtrakul P., Prachayawarakorn S. (2011). Effects of cooking, drying and pretreatment methods on texture and starch digestibility of instant rice. J. Food Eng..

[61] Yang L., Sun Y.H., Liu Y., Mao Q., You L.X., Hou J.M., Ashraf M.A. (2016). Effects of leached amylose and amylopectin in rice cooking liquidon texture and structure of cooked rice. Braz. Arch. Biol. Technol..

[62] Chandi G.K., Sogi D.S. (2008). Characterization of traditional (Basmati 370) and developed (Pusa Basmati 1) basmati rice. Int. J. Food Prop..

